# Inhibition of HIF prolyl-4-hydroxylases by FG-4497 Reduces Brain Tissue Injury and Edema Formation during Ischemic Stroke

**DOI:** 10.1371/journal.pone.0084767

**Published:** 2014-01-07

**Authors:** Stefan Reischl, Lexiao Li, Gail Walkinshaw, Lee A. Flippin, Hugo H. Marti, Reiner Kunze

**Affiliations:** 1 Institute of Physiology and Pathophysiology, University of Heidelberg, Heidelberg, Germany; 2 FibroGen, Inc., San Francisco, California, United States of America; Hôpital Robert Debré, France

## Abstract

Ischemic stroke results in disruption of the blood-brain barrier (BBB), edema formation and neuronal cell loss. Some neuroprotective factors such as vascular endothelial growth factor (VEGF) favor edema formation, while others such as erythropoietin (Epo) can mitigate it. Both factors are controlled by hypoxia inducible transcription factors (HIF) and the activity of prolyl hydroxylase domain proteins (PHD). We hypothesize that activation of the adaptive hypoxic response by inhibition of PHD results in neuroprotection and prevention of vascular leakage. Mice, subjected to cerebral ischemia, were pre- or post-treated with the novel PHD inhibitor FG-4497. Inhibition of PHD activity resulted in HIF-1α stabilization, increased expression of VEGF and Epo, improved outcome from ischemic stroke and reduced edema formation by maintaining BBB integrity. Additional *in vitro* studies using brain endothelial cells and primary astrocytes confirmed that FG-4497 induces the HIF signaling pathway, leading to increased VEGF and Epo expression. In an *in vitro* ischemia model, using combined oxygen and glucose deprivation, FG-4497 promoted the survival of neurons. Furthermore, FG-4497 prevented the ischemia-induced rearrangement and gap formation of the tight junction proteins zonula occludens 1 and occludin, both in cultured endothelial cells and in infarcted brain tissue *in vivo*. These results indicate that FG-4497 has the potential to prevent cerebral ischemic damage by neuroprotection and prevention of vascular leakage.

## Introduction

Stroke is one of the most relevant causes of death in the western world and responsible for enormous economic costs caused by illness. However, an effective clinical therapy, initiated in the first hours after stroke, with the potential to attenuate the consequences of an ischemic insult is currently not available. Moreover, patients suffering from stroke or transient ischemic attacks incur an increased risk of a recurrent stroke within days or weeks of their sentinel event. Similarly, patients with cardiovascular diseases like intracranial artery stenosis or atrial fibrillation are at high risk of stroke [1]. Thus, development of new prophylactic therapies such as delivery of potent neuroprotective agents before the onset of cerebral ischemia would help to reduce stroke burden in these high risk patients. Ischemic stroke causes acute neuronal death by oxygen and nutrient depletion and initiates blood-brain barrier (BBB) malfunction. Dysfunction of the BBB results from impairment of interendothelial tight and adherens junctions required to maintain BBB integrity. BBB hyperpermeability not only favors the formation of vasogenic cerebral edema, but also promotes infiltration of circulating leukocytes. Hence, death of compromised neurons in the infarct border zone is triggered, thus expanding the initial infarct core [2], [3], [4]. Nevertheless, decline of the cellular oxygen tension during ischemic stroke also promotes an endogenous adaptive response accompanied by up-regulation of various cytoprotective factors to attenuate the ischemic injury [5]. Among them vascular endothelial growth factor (VEGF) and erythropoietin (Epo) originally identified as master regulators of angiogenesis and erythropoiesis, respectively, have also been shown to promote neuronal survival upon cerebral ischemia through induction of anti-apoptotic pathways [6], [7], [8], [9], [10]. Moreover, treatment with recombinant human Epo was shown to preserve the BBB integrity in mice undergoing ischemic stroke [11], [12]. The majority of these oxygen-dependent regulated genes including VEGF and Epo are target genes of hypoxia-inducible transcription factors (HIFs), heterodimeric proteins consisting of an α (1α, 2α or 3α) and a β subunit [13]. HIF activity is primarily affected via oxygen-dependent regulation of HIF-α protein stability. Under normoxic conditions, the α subunit is hydroxylated on conserved proline residues, which results in recruitment of the von Hippel-Lindau protein E3 ubiquitin ligase and immediate proteasomal degradation of HIF-α [13]. This prolyl hydroxylation is mediated through a family of prolyl-4-hydroxylase domain (PHD) proteins, whose activity is dependent on molecular oxygen, ferrous iron and 2-oxoglutarate. In contrast, when the cellular oxygen level declines during hypoxia or ischemia, PHDs are less active. As a result, unhydroxylated HIF-α accumulates, dimerizes with the β subunit, and subsequently enters the nucleus to induce expression of HIF responsive genes [13]. The HIF-PHD family members (PHD1-3) are expressed in essentially all mammalian tissues studied; however, the expression level of each isoenzyme varies. PHD2 is uniformly expressed in most of the tissues, while the highest PHD1 and PHD3 mRNA expression levels have been detected in the testis and heart, respectively [14]. Previously, we demonstrated that in adult mouse brain PHD2 possesses a higher mRNA expression level as compared to the other isoenzymes [15]. Moreover, *in vitro* studies revealed that PHD2 may act preferentially on HIF-1α than on HIF-2α, whereas PHD3 has an opposite selectivity [14]. Genetic knockout studies in mice showed that PHD2 deficiency leads to embryonic lethality, while PHD1 and PHD3 null mice were viable and apparently normal, substantiating the key role of PHD2 as the master regulator of the hypoxic response [14].

Thus, application of low-molecular weight BBB permeable PHD inhibitors, before or immediately after onset of cerebral ischemia, could be a promising clinical strategy to reduce brain tissue damage. Accordingly, the present study was designed to analyze whether pre- and post-treatment with the novel PHD inhibitor FG-4497 alleviates cerebral tissue injury in mice suffering from ischemic stroke.

## Materials and Methods

### Middle Cerebral Artery Occlusion (MCAO)

All experiments were performed using male C57BL/6 mice (8 to 12 weeks old). Animals were maintained at the animal facility of the University of Heidelberg. All animal procedures were approved by the local animal welfare committee (Regierungspräsidium Karlsruhe). Mice were anaesthetized by a mixture containing 4% halothane, 70% N_2_O, and remainder O_2_, and were maintained by reducing the halothane concentration to 1.0–1.5%. Body temperature was maintained at 37°C using a temperature controlled heating pad. To induce permanent focal cerebral ischemia a skin incision was made between the lateral corner of the left eye and the ear followed by removal of the temporal muscle through electrocoagulation. Then, a hole was drilled through the skull to expose the distal MCA, which was occluded by microbipolar coagulation. The skin was sutured after surgery and the mice were kept for 7 days. For transient focal cerebral ischemia a laser-Doppler flowmetry (LDF) probe (Perimed Instruments, Rommerskirchen, Germany) was positioned 1.5 mm posterior and 3 mm lateral from bregma. Then, a 7–0 silicon rubber-coated monofilament (Doccol Corporation, Redlands, USA) was introduced in the internal carotid artery and pushed toward the MCA until a drop in regional cerebral blood flow (rCBF) below 30% from baseline was documented by LDF. After 60 min occlusion, reperfusion was allowed for 24 h. Subsequently, animals were killed by decapitation, brains were removed and embedded into Tissue-Tek (Sakura Finetek, Staufen, Germany) for histological analyses. From each brain, 24 coronal cryosections (10 µm thick each; 0.4 mm apart) were prepared and submitted to Nissl staining. Healthy tissue appears dark while infarcted tissue appears light (cresyl violet-deficient) [16]. The total infarct volume was calculated as the summation of the total infarct area of each section multiplied by the distance between each section. The area of each hemisphere was measured by ImageJ software. To calculate the infarct area of each section the following equation was applied: I = (CD+CT−IT), where I = infarct area in mm^2^, CD = cresyl violet-deficient area in mm^2^, CT = total area of the contralateral hemisphere in mm^2^, IT = total area of the ipsilateral hemisphere in mm^2^. Thus, the total infarct volume in mm^3^ = ΣI*0.4, where 0.4 = the distance between each section in mm. This equation was used to correct for the increase in volume of the ipsilateral hemisphere due to vasogenic edema-induced swelling [15]. Accordingly, following equation was used to calculate the edema area of each section: E = (IT–CT), where E = edema area in mm^2^, and the total edema volume in mm^3^ = ΣI*0.4. For biochemical analyses anaesthetized animals were transcardially perfused with PBS (2 ml/min) for 5 min, brains were removed and shock frozen in liquid nitrogen.

### FG-4497 Administration *in vivo*

FG-4497 (FibroGen, San Francisco, USA) was dissolved according to manufacturer’s instructions (100 mg FG-4497 to 9.675 ml of 5% Dextrose and 325 µl of 1 N NaOH), and was injected intraperitoneally (injection volume 0.2–0.25 ml) as a single dose of 100 mg/kg. Control mice received 0.2–0.25 ml vehicle solution (0.9% NaCl) through intraperitoneal application.

### Isolation of Primary Murine Astrocytes

C57BL/6 neonatal mice (P0) were sacrificed by decapitation and brains were dissected. Subsequent to precautious removal of meningeal plexus, meninges and cerebella, brains were mechanically homogenized in DMEM medium (Invitrogen, Darmstadt, Germany) supplemented with 20% FBS, 100 units/ml penicillin, 100 µg/ml streptomycin and 2 mM L-glutamin (Invitrogen). Then, the cell suspension was filtered consecutively through sterile cell strainer of 150 µm and 100 µm mesh size, respectively followed by centrifugation at 800 rpm for 10 min. The pellet was resuspended in culture medium and cells were plated on culture flasks. During culturing for 3–4 weeks medium was replaced two times per week and FBS concentration was reduced stepwise from 20% to 5%. Then, microglial cells and oligodendrocytes were removed from mixed glia cultures by shaking flasks at 200 rpm for 2 hours.

### Cell Culture

Primary murine astrocytes, the murine cerebrovascular endothelial cell line bEnd.3 [17] and the mouse hippocampal neuronal cell line HT-22 [18] were seeded at 10,000 cells/cm^2^ in DMEM containing 100 units/ml penicillin, 100 µg/ml streptomycin, 2 mM L-glutamin and 5% or 10% FBS, respectively and cultured at 37°C in a humidified incubator with 5% CO_2_ in air. Upon reaching confluence, cells were treated with either dimethyloxalylglycine (DMOG; Cayman Chemical Co., Ann Arbor, USA) or FG-4497 (both dissolved in DMSO). During treatment FBS concentration was reduced to 1% as FG-4497 is reported to have high binding affinity to serum proteins [19].

Oxygen-glucose deprivation (OGD) was performed by culturing cells in an incubator flooded with humidified gas mixture consisting of 5% CO_2_ and 95% N_2_ (<1% O_2_) at 37°C. Normal culture media was replaced with glucose-free DMEM supplemented with ≤0.2% FBS.

### Analysis of Cell Viability

Cellular viability was determined using the Live/Dead assay (Invitrogen) according to manufacturer’s instructions. Cells were incubated with 10 µM Calcein-AM and 10 µM EthD-1 for 45 min to stain living cells (Calcein-AM positive) and dead cells (EthD-1 positive). Stained cells were visualized using an Olympus BX50 fluorescence microscope, and digital images were acquired with a Leica DC 500.

### Immunofluorescence Analyses

Coronal cryosections (10 µm in thickness) and cell monolayers were fixed with zinc-based fixative for 30 min and were permeabilized with 0.5% saponin in PBS for 15 min. Then, brain slices and cells were incubated for 30–60 min in blocking buffer consisting of 10% goat serum (Dianova, Hamburg, Germany) in PBST (0.1% Tween-20 in PBS) or 1% BSA in PBS, respectively. Subsequently, slices and cells were incubated overnight at 4°C with antibodies against occludin (1%; Invitrogen) or ZO-1 (1%; Invitrogen) followed by incubation with appropriate Cy3-conjugated secondary antibodies (0.5%; Dianova) for 1 h. All antibodies were diluted in LowCross-Buffer (Candor, Wangen, Germany). Slices and cells were incubated for 10 min with 0.02% DAPI (Invitrogen) in PBS to stain nuclei. Stained sections and cells on glass slides were then embedded in Mowiol mounting medium (Sigma-Aldrich, Steinheim, Germany). Fluorescence staining was recorded using an Olympus BX50 microscope with a Leica DC 500 camera. For confocal microscopy analysis, an Olympus IX81 confocal microscope and camera equipped with a 60×objective was used.

### RNA Isolation and Real-time PCR

Total RNA from cells or brain tissue was isolated using the TRI reagent (Invitrogen) according to manufacturer’s instructions. For digestion of residual DNA, 10 µg of total RNA was incubated in a 25 µl reaction mix containing 1×DNase-buffer, 40 U RNasin and 1 U DNase (Promega, Mannheim, Germany) for 30 min at 37°C. Reverse transcription (1 µg RNA) was carried out with Access RT-PCR Kit (Promega). Real-time PCR was performed using the QuantiTect SYBR Green PCR Kit (Qiagen, Hilden, Germany) corresponding to manufacturer’s instructions. The housekeeping gene ribosomal protein S12 (*Rps12*) was used as control. The following primers (MWG Biotech, Ebersberg, Germany) were used: *Epo* fwd CCACCCTGCTGCTTTTACTC, *Epo* rev CTCAGTCTGGGACCTTCTGC, *Phd1* fwd GCTAGGCTGAGGGAGGAAGT, *Phd1* rev CCCCAAGTTGTCCTTGA, *Phd2* fwd TTGCTGACATTGAACCCAAA, *Phd2* rev GGCAACTGAGAGGCTGTAGG, *Phd3* fwd GCTATCCAGGAAATGGGACA, *Phd3* rev TGGCGTCCCAATTCTTATTC, *Rps12* fwd GAAGCTGCCAAAGCCTTAGA, *Rps12* rev AACTGCAACCAACCACCTTC, *Vegf* fwd GTACCTCCATGCCAAGT, *Vegf* rev ACTCCAGGGCTTCATCGTTA.

### Protein Extraction

Cells were lysed in homogenization buffer containing 20 mM Tris (pH 7.6), 250 mM NaCl, 1 mM EDTA, 1 mM EGTA, 1% Triton X-100, 0.5% Nonidet P-40, 1 mM DTT, 1 mM PMSF, 1% protease inhibitor cocktail and 4 mM Na_3_VO_4_ (all from Sigma-Aldrich). Following incubation on ice for 15 min, samples were centrifuged at 14,000 g and 4°C for 15 min and the supernatants (cellular protein extract) were frozen at −80°C until use.

Brain tissue was mechanically homogenized in 750 µl of buffer A containing 10 mM Hepes (pH 7.9), 10 mM KCl, 0.1 mM EDTA, 0.1 mM EGTA, 1 mM DTT, 1 mM PMSF, 1% protease inhibitor cocktail, 1% Nonidet P-40 and 2 mM Na_3_VO_4_. After 15 min incubation on ice, samples were centrifuged at 850 g and 4°C for 10 min. Pellets were resuspended in 1 ml of buffer B (buffer A without Nonidet P-40) and incubated on ice for 15 min. Then, 75 µl of 10% Nonidet P-40 was added, the suspension was mixed thoroughly for 10 s followed by centrifugation at 14,000 g and 4°C for 1 min. Pellets were resuspended in 100 µl buffer C consisting of 20 mM Hepes (pH 7.9), 0.4 mM NaCl, 0.1 mM EDTA, 0.1 mM EGTA, 1 mM DTT, 1 mM PMSF, 1% protease inhibitor cocktail and 2 mM Na3VO_4_, and were incubated on ice for 30 min with gentle shaking. After a final centrifugation at 14,000 g and 4°C for 10 min, the supernatants (nuclear protein extract) were snap-frozen in liquid nitrogen.

### Immunoblotting

Proteins (25 µg) were run on a SDS-polyacrylamide gel and then transferred onto a nitrocellulose membrane. The TATA binding protein (TBP) or β-actin were used as loading control. The membrane was incubated overnight at 4°C with primary antibodies against HIF-1α (0.2%; Novus Biologicals, Cambridge, UK), HIF-2α (0.5%; R&D Systems, Wiesbaden, Germany), PHD3 (0.1%; Novus Biologicals), β-actin (0.2%; Sigma-Aldrich) or TBP (0.1%; Abcam, Cambridge, UK) followed by incubation with appropriate HRP-conjugated secondary antibodies (0.02%; Dianova; Thermo Fisher Scientific, Bonn, Germany) for 1 h. All antibodies were diluted in blocking buffer consisting of 5% nonfat dry milk in TBST (0.1% Tween-20 in TBS). ECL Western blot detection reagents (GE Healthcare, Freiburg, Germany) were used for protein detection.

### Statistical Analysis

The results are presented as mean ± standard error of the mean (SEM). Unpaired two-tailed t-test or one-way analysis of variance (ANOVA) combined with Bonferroni post-test were applied to determine statistical significance. Data plotting and statistical analyses were done in Prism 5 (GraphPad Software, San Diego, USA).

## Results

### FG-4497 Induces the HIF Signaling Pathway in Endothelial Cells and Astrocytes

**Figure 1:**
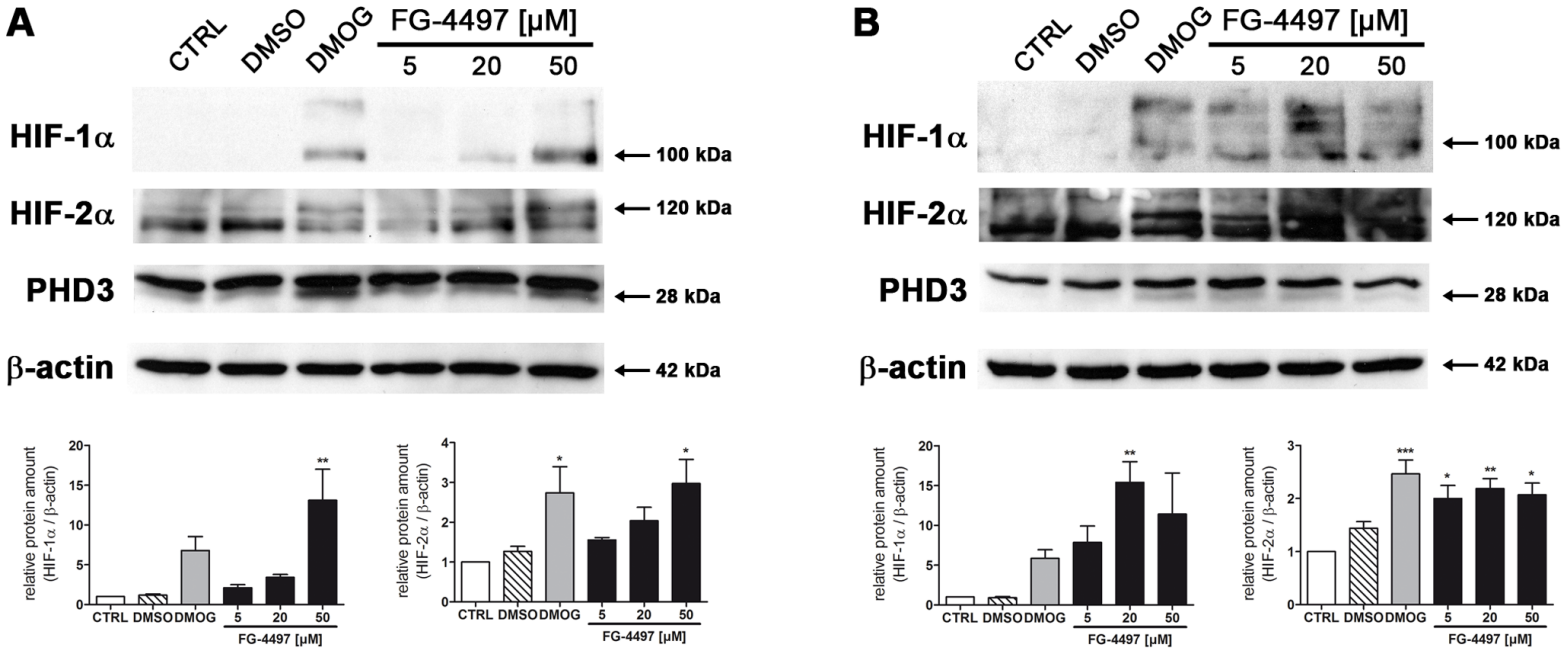
FG-4497 increases HIF-α and PHD3 protein abundance in endothelial cells and astrocytes. Mouse cerebrovascular bEnd.3 cells (**A**) and primary murine astrocytes (**B**) were treated with either 1 mM DMOG or FG-4497 (5, 20 and 50 µM) for 6 hours. As DMOG and FG-4497 were dissolved in DMSO, 0.2% DMSO was used as a control. Then, cellular proteins were isolated and HIF-1α, HIF-2α and PHD3 abundance was quantified by Western blot. Values are normalized to β-actin and expressed as fold change to CTRL (control = non-treated cells). Significant differences determined by one-way ANOVA combined with Bonferroni post-test are indicated with *(*p*<0.05), **(*p*<0.01) or ***(*p*<0.001). *N* = 3.

In endothelial cells and astrocytes, both cell types building up the BBB *in vivo*, the potential of FG-4497 to induce the HIF signaling pathway was analyzed, and compared to the well-known pan-hydroxylase inhibitor DMOG. Treatment with FG-4497 stabilized HIF-1α protein in both primary murine astrocytes and the murine cerebrovascular endothelial cell line bEnd.3 (Fig. 1). FG-4497 exceeded the capacity of DMOG to protect HIF-1α against proteolytic degradation (Fig. 1). Additionally to HIF-1α, FG-4497 and DMOG also significantly increased the protein abundance of HIF-2α in endothelial cells and astrocytes (Fig. 1). In both cell types expression of the HIF target gene PHD3 on protein level was induced upon treatment with DMOG or FG-4497 (Fig. 1).

**Figure 2:**
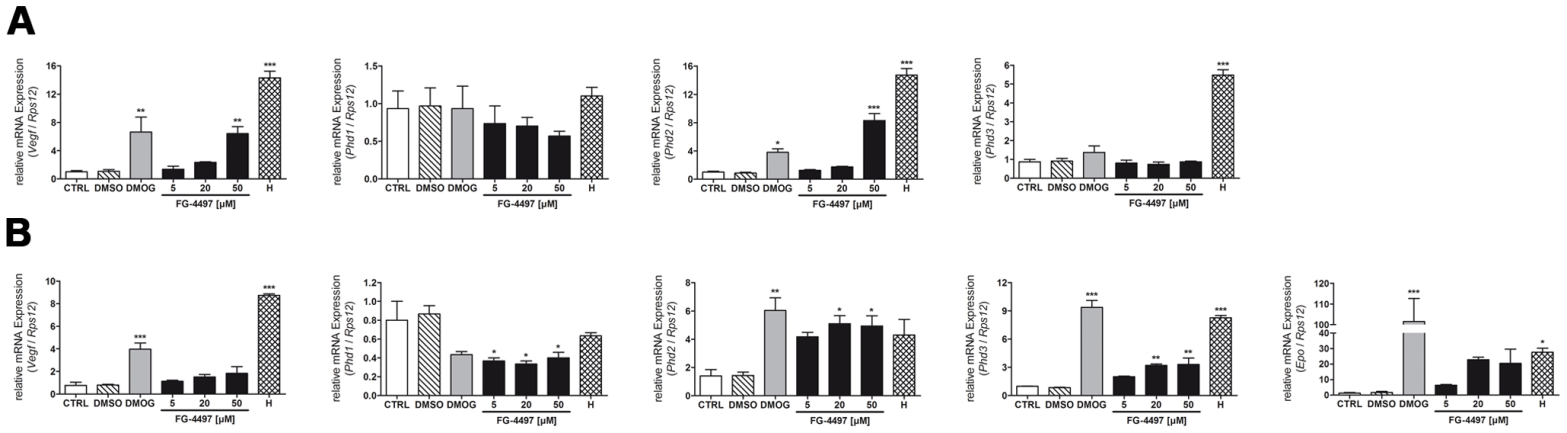
FG-4497 induces HIF target gene expression in endothelial cells and astrocytes. Mouse cerebrovascular bEnd.3 cells (**A**) and primary murine astrocytes (**B**) were treated with either 1 mM DMOG or FG-4497 (5, 20 and 50 µM) or were exposed to hypoxia (1% O_2_) for 12 hours. Subsequently, RNA was isolated and gene expression was analyzed by real-time PCR. Values are normalized to *Rps12* and expressed as fold change to CTRL. Significant differences determined by one-way ANOVA combined with Bonferroni post-test are indicated with *(*p*<0.05), **(*p*<0.01) or ***(*p*<0.001). *N* = 3.

Moreover, endothelial transcription of the HIF target genes VEGF and PHD2 was significantly increased upon treatment with either FG-4497 or DMOG (Fig. 2A). In astrocytes FG-4497 application led to up-regulation of the HIF-targeted genes VEGF, Epo, PHD2 and PHD3. However, transcriptional induction of VEGF, Epo and PHD3 was lower as compared to DMOG (Fig. 2B). Of note, Epo mRNA in (non-)treated endothelial cells was below the detection limit of our real-time PCR assay. Notably, both cell types responded to FG-4497 with transcriptional down-regulation of PHD1 (Fig. 2).

### FG-4497 Sustains Subcellular Localization of Endothelial Tight Junction-associated Proteins during Ischemic Conditions *in vitro*

**Figure 3:**
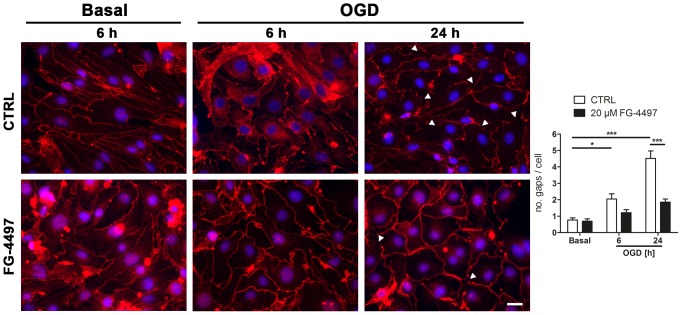
FG-4497 prevents subcellular delocalization of ZO-1 in OGD-stressed endothelial cells. Mouse cerebrovascular bEnd.3 cells were exposed to OGD in the absence or presence of 20 µM FG-4497 for 6 and 24 hours or were treated with 20 µM FG-4497 for 6 hours under basal conditions. Immunofluorescent staining was used to visualize the subcellular localization of ZO-1. Cell membrane regions not immunoreactive for ZO-1 (gaps; denoted by white arrowheads) were quantified in five different randomly chosen microscopic fields for each condition per experiment. Significant differences determined by one-way ANOVA combined with Bonferroni post-test are indicated with *(*p*<0.05) or ***(*p*<0.001). Scale bar = 20 µm. *N* = 4.

Integrity of the BBB is highly dependent on interendothelial tight junctions (TJ) consisting of integral and peripheral membrane proteins such as occludin, claudin-5 and Zonula occludens (ZO)-1 [4]. Here, we show that ischemic conditions *in vitro* caused subcellular delocalization of ZO-1 in brain endothelial cells (Fig. 3). In contrast, FG-4497 treated endothelial cells sustained the membrane localization of ZO-1 also upon ischemic stress (Fig. 3). Notably, FG-4497 had no effect on TJ localization under basic conditions (Fig. 3).

**Figure 4:**
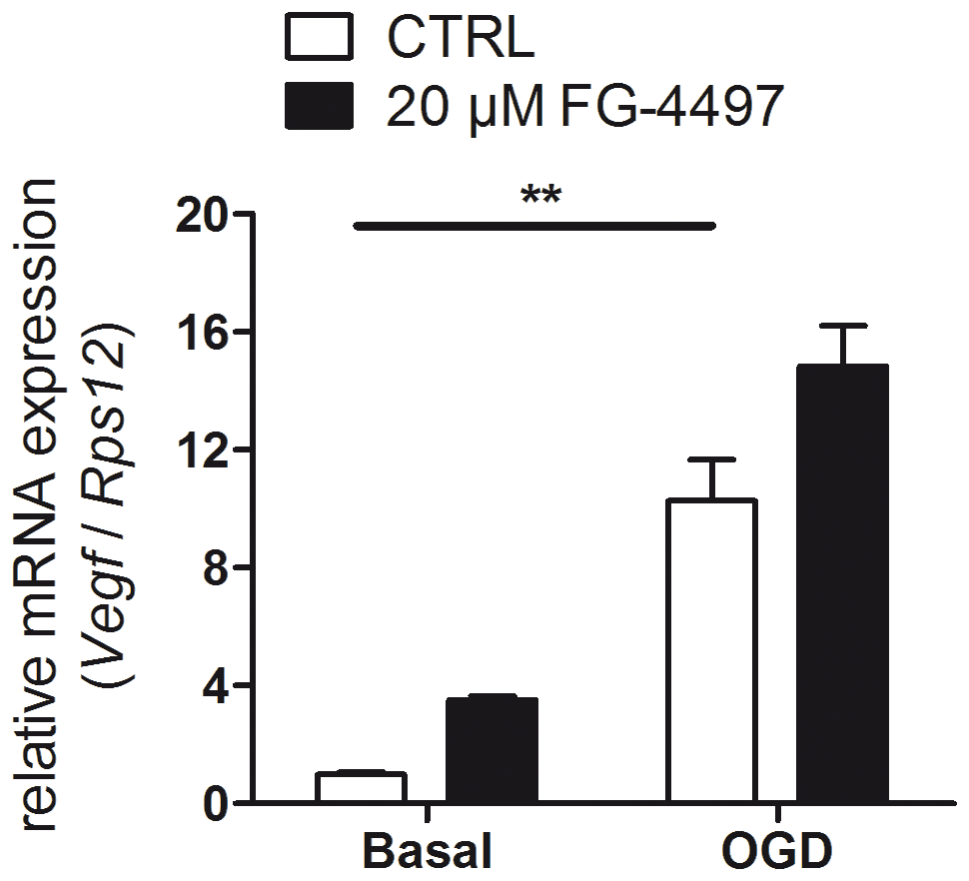
Effect of FG-4497 on the VEGF expression in endothelial cells exposed to ischemic conditions. Mouse cerebrovascular bEnd.3 cells were treated with 20 µM FG-4497 for 6 hours under OGD or basal conditions. Subsequently, RNA was isolated and gene expression was analyzed by real-time PCR. Values are normalized to *Rps12* and expressed as fold change to untreated cells cultured under non-ischemic conditions. Significant differences determined by one-way ANOVA combined with Bonferroni post-test are indicated with **(*p*<0.01). *N* = 3.

Under basal conditions endothelial cells treated with FG-4497 showed higher VEGF expression as compared to control cells (Fig. 4). OGD potentially stimulated VEGF production in both the absence and presence of the PHD inhibitor, such that expression of VEGF in FG-4497 treated endothelial cells exposed to OGD was not significantly different as compared to non-treated cells exposed to OGD (Fig. 4).

### FG-4497 Attenuates Neuronal Cell Death during Ischemic Conditions *in vitro*

**Figure 5:**
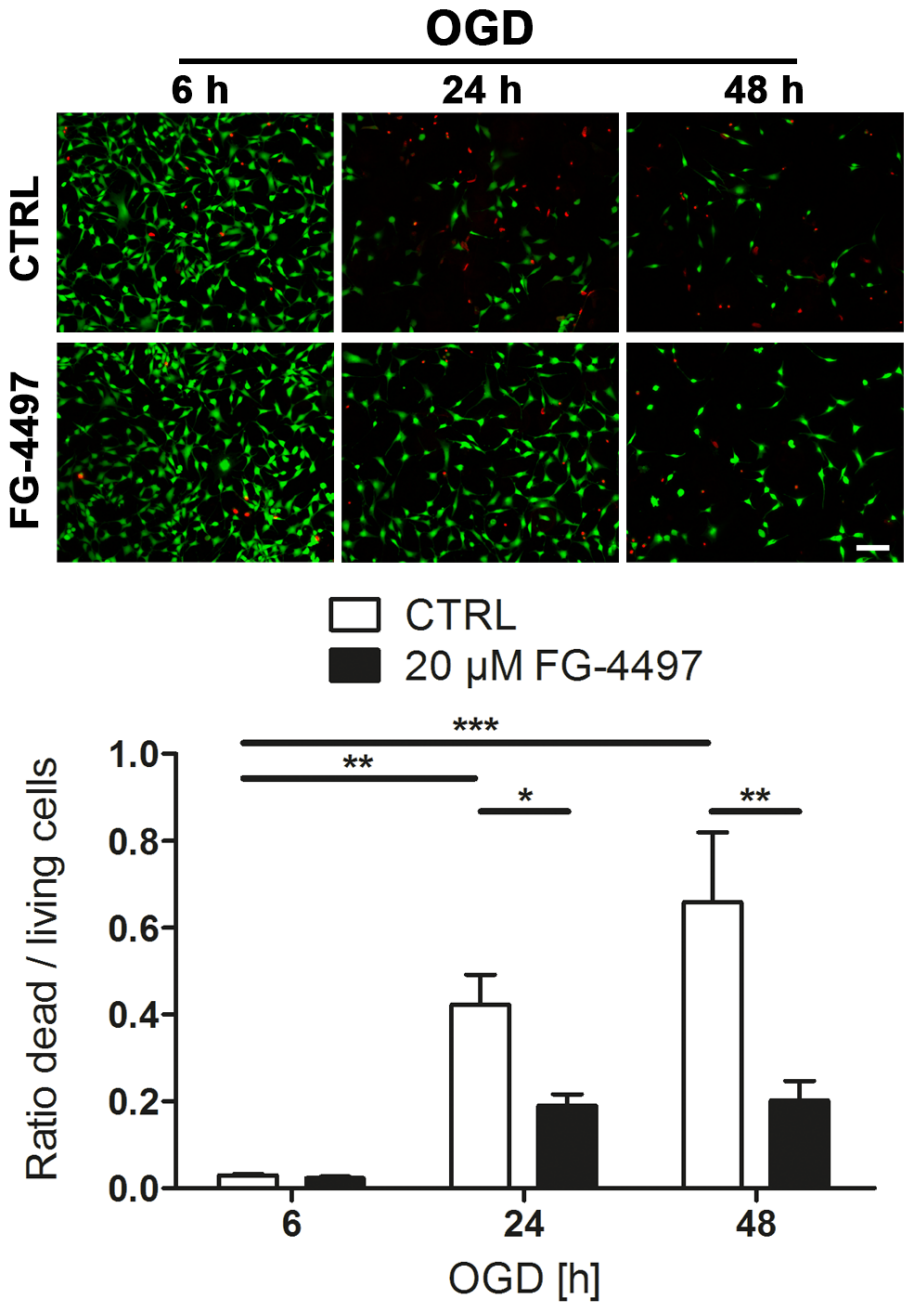
FG-4497 attenuates neuronal cell death under ischemic conditions. Mouse hippocampal HT-22 neuronal cells were exposed to OGD conditions in the absence or presence of 20 µM FG-4497 for 6, 24 and 48 hours. Cell death/survival was determined by using the Live/Dead assay. Dead (red) and living (green) cells were quantified in three randomly chosen microscopic fields for each condition per experiment, and the ratio dead/living cells was calculated. Significant differences determined by one-way ANOVA combined with Bonferroni post-test are indicated with *(*p*<0.05), **(*p*<0.01) or ***(*p*<0.001). Scale bar = 100 µm. *N* = 6.

To evaluate the direct neuroprotective potential of FG-4497 mouse hippocampal neuronal HT-22 cells were exposed to *in vitro* ischemic conditions (OGD) in the presence or absence of the drug. Viability of HT-22 cells was strongly impaired upon OGD (Fig. 5). In the presence of FG-4497, however, survival of neurons was significantly improved (Fig. 5), indicative of a direct neuroprotective mechanism.

### FG-4497 Promotes the HIF Signaling Pathway in Cerebral Tissue *in vivo*

**Figure 6:**
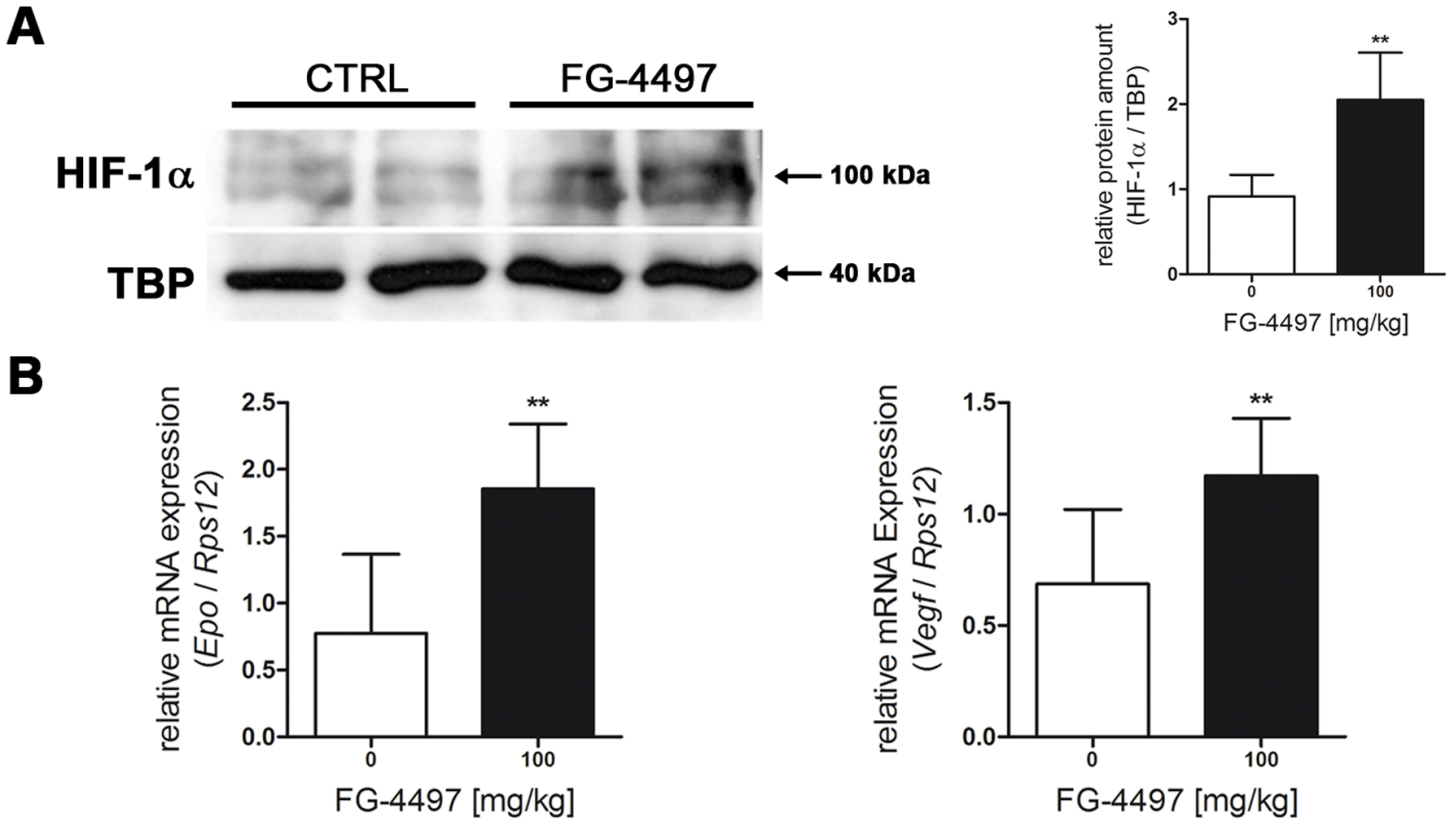
FG-4497 promotes the HIF signaling pathway in cerebral tissue *in vivo*. 100/kg FG-4497 or an equal volume of 0.9% NaCl was applied intraperitoneally to adult C57BL/6 mice. After 6 hours brains were removed, nuclear proteins and RNA were prepared from cerebral tissue and Western blot (**A**) and real-time PCR (**B**) was performed, respectively. Values are normalized to TBP (protein) or *Rps12* (RNA) and expressed as fold change to vehicle treated animals. Significant differences determined by unpaired two-tailed t-test are indicated with **(*p*<0.01). *N* = 5–8 (per group).

To address the effect of FG-4497 *in vivo,* the drug (100 mg/kg) was administered intraperitoneally to mice. FG-4497 administration increased HIF-1α protein abundance in cerebral tissue (Fig. 6A). Accordingly, mRNA expression of VEGF and Epo in brain tissue was significantly up-regulated following FG-4497 treatment (Fig. 6B).

### FG-4497 Treatment Reduces Tissue Damage and Vasogenic Edema Formation Provoked by Ischemic Stroke

**Figure 7:**
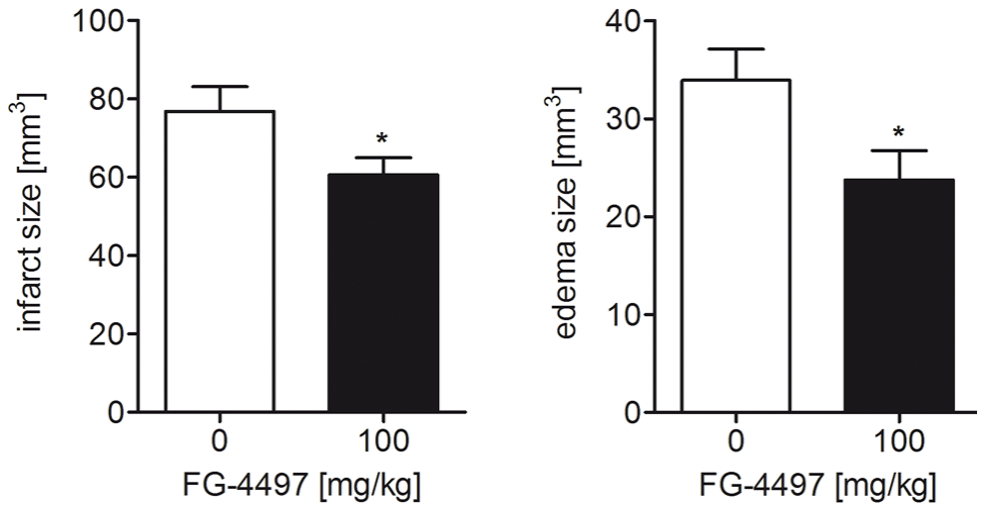
FG-4497 pre-treatment decreases infarct size and the formation of vasogenic edema upon transient cerebral ischemia. 100/kg FG-4497 or an equal volume of 0.9% NaCl was applied intraperitoneally to adult C57BL/6 mice. After 6 hours mice underwent 60 min of MCAO followed by 24 hours reperfusion. Subsequently, brains were removed and from each brain, 24 coronal cryosections (10 µm thick each; 0.4 mm apart) were prepared and submitted to cresyl violet staining for quantification of the infarct and edema size. Significant differences determined by unpaired two-tailed t-test are indicated with *(*p*<0.05). *N* = 8–9 (per group).

The effect of FG-4497 was then evaluated *in vivo* in two stroke models. In a first setting mice were pre-treated with single dose of FG-4497 (100 mg/kg) 6 hours before transient MCAO. These animals showed a significantly reduced infarct size and decreased formation of vasogenic edema after 24 hours reperfusion as compared to vehicle treated mice (Fig. 7).

**Figure 8:**
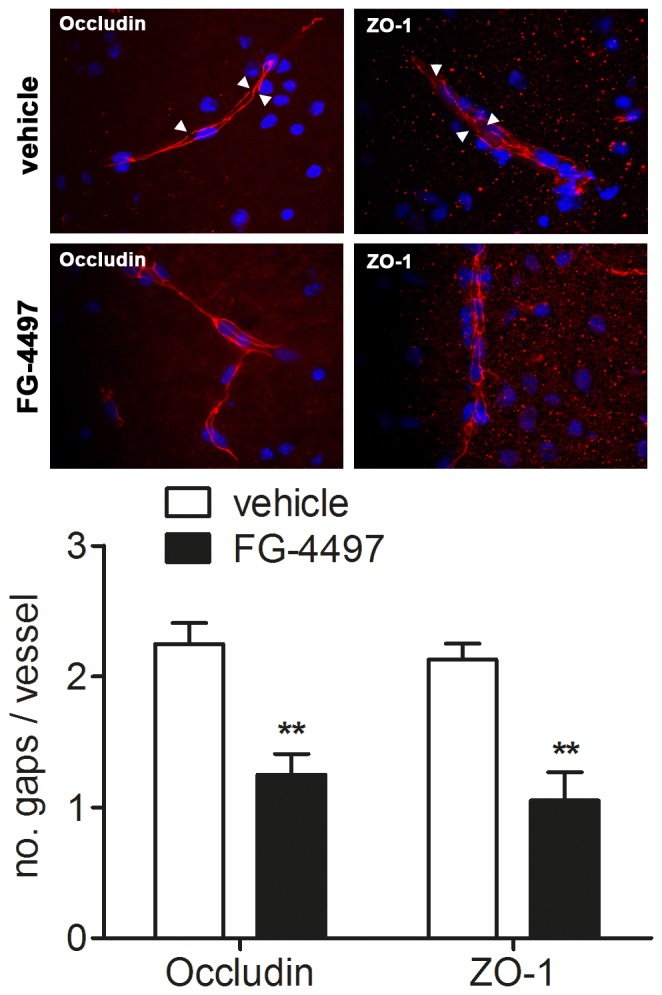
FG-4497 sustains subcellular localization of occludin and ZO-1 in cerebral blood capillaries upon ischemic stroke. 100/kg FG-4497 or an equal volume of 0.9% NaCl was applied intraperitoneally to adult C57BL/6 mice. After 6 hours mice underwent 60 min of MCAO followed by 24 hours reperfusion. Subsequently, brains were removed, coronal cryosections were prepared and immunofluorescent detection of occludin and ZO-1 was performed. Disruptions (gaps; denoted by white arrowheads) of the regular occludin and ZO-1 localization pattern, respectively were counted in at least three randomly chosen blood vessels localized within the peri-infarct region per animal, and the number of gaps per vessel was calculated. Significant differences determined by unpaired two-tailed t-test are indicated with **(*p*<0.01). *N* = 4 (per group).

Immunofluorescent analysis of the TJ proteins occludin and ZO-1 within infarcted brain tissue revealed a pronounced disruption of their regular subcellular localization along the endothelial cell membrane in blood vessels of vehicle treated mice (Fig. 8). By contrast, in animals receiving FG-4497 the regular membrane-associated localization of occludin and ZO-1 was maintained, and gap formation was significantly reduced (Fig. 8).

**Figure 9:**
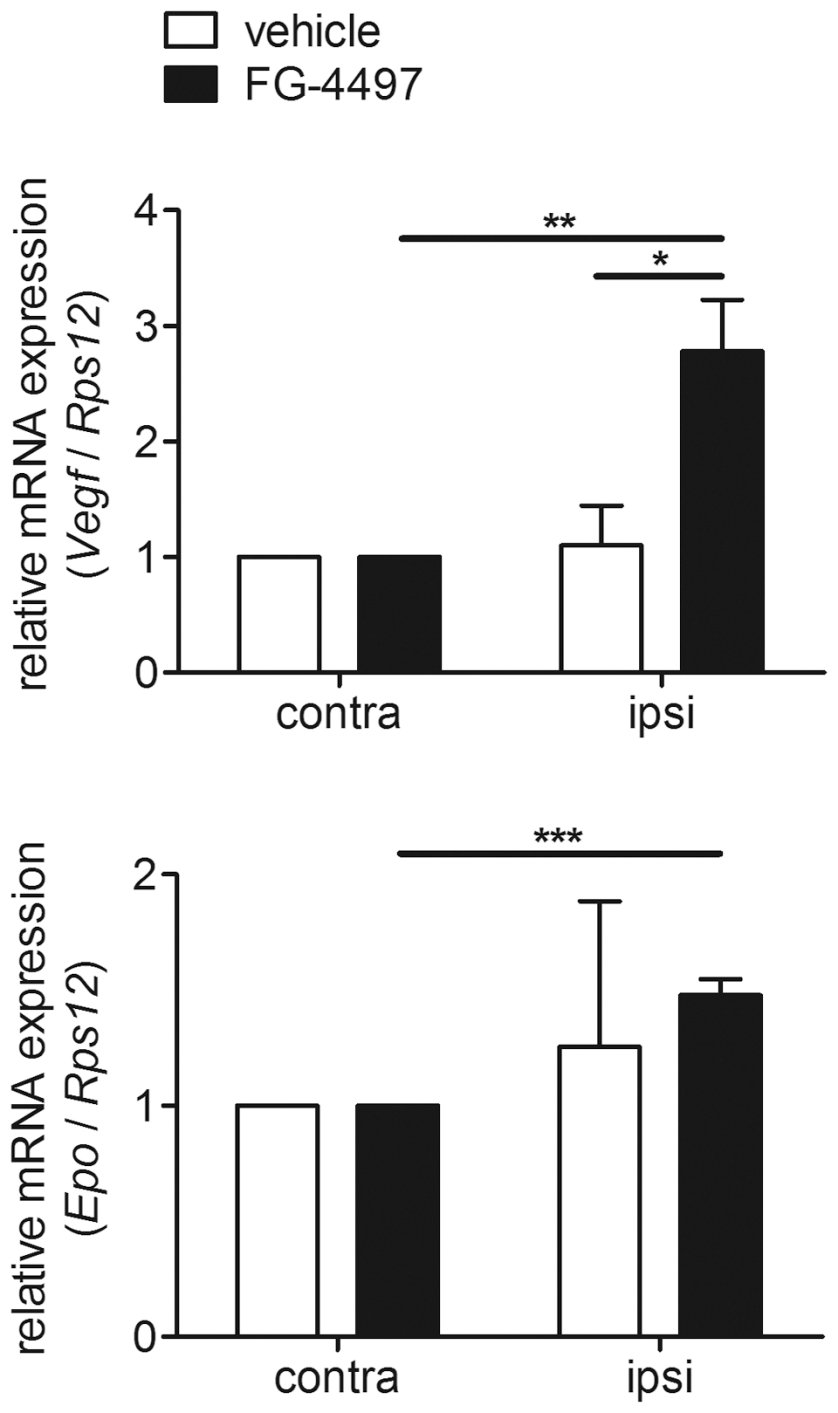
FG-4497 increases VEGF and Epo expression in infarcted brain tissue. 100/kg FG-4497 or an equal volume of 0.9% NaCl was applied intraperitoneally to adult C57BL/6 mice. After 6 hours mice underwent 60 min of MCAO followed by 24 hours reperfusion. Then, brains were removed, RNA was prepared from non-ischemic contralateral and ischemic ipsilateral hemisphere and real-time PCR was performed. Values are normalized to *Rps12* and expressed as fold change to vehicle treated animals. Significant differences determined by unpaired two-tailed t-test are indicated with *(*p*<0.05), **(*p*<0.01) or ***(*p*<0.001). *N* = 3–4 (per group).

During the post-stroke reperfusion period mRNA expression of the HIF target genes VEGF and Epo within the non-ischemic contralateral hemisphere did not differ between FG-4497 and vehicle treated animals. Nevertheless, when comparing VEGF and Epo expression in contralateral versus ischemic ipsilateral hemisphere a significant up-regulation of both genes was determined in mice receiving FG-4497 but not in vehicle treated animals (Fig. 9).

**Figure 10:**
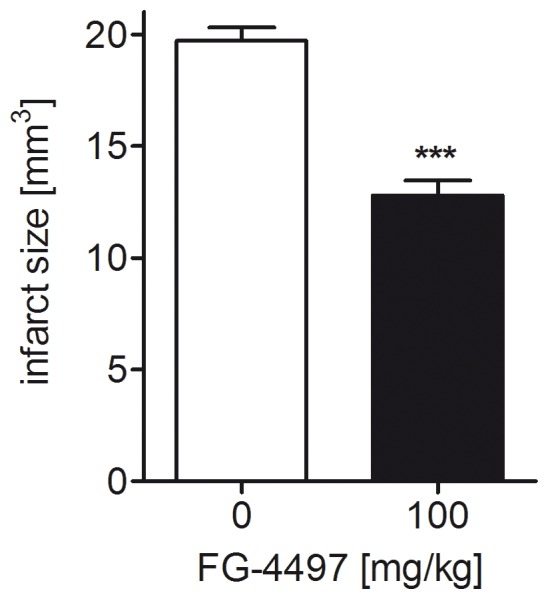
FG-4497 post-treatment reduces infarct size upon permanent cerebral ischemia. Adult C57BL/6 mice underwent permanent MCAO followed by intraperitoneal application of 100 mg/kg FG-4497 or an equal volume of 0.9% NaCl 1 hour later. 7 days after induction of permanent cerebral ischemia, brains were removed and from each brain, 24 coronal cryosections (10 µm thick each; 0.4 mm apart) were prepared and submitted to cresyl violet staining for quantification of the infarct size. Significant differences determined by unpaired two-tailed t-test are indicated with ***(*p*<0.001). *N* = 6 (per group).

As development of effective therapies initiated during the early phase after ischemic stroke is of highest clinical importance, we evaluated in a second set of experiments the capacity of FG-4497 to diminish tissue injury when applied one hour after stroke. We could show that a single post-treatment with the PHD inhibitor FG-4497 is sufficient to significantly decrease tissue damage at 7 days after permanent MCAO (Fig. 10). Taken together, as reduced brain injury was detected both in the acute (1 day; Fig. 7) and the sub-acute phase (7 days; Fig. 10) upon cerebral ischemia, our results suggest that FG-4497 may improve the long-term outcome from stroke.

## Discussion

The neuroprotective effect of VEGF has been demonstrated in many studies [8], [9], [10], [20] making it an attractive candidate for stroke prevention and therapy. However, the use of VEGF is prevented by its promotion of vascular leakage resulting in brain edema and further cerebral damage. Thus, other strategies to protect the brain from ischemic damage are needed. We recently showed that neuronal deletion of PHD2 in mice is sufficient to confer neuroprotection after stroke without inducing vascular leakage despite VEGF up-regulation [16]. Thus, PHD inhibition might be a promising approach to prevent and treat cerebral ischemia. Our data now show that this approach is adaptable to the therapeutic situation.

### The PHD Inhibitor FG-4497 Improves Stroke Outcome

Our data clearly indicate that systemic pre- as well as post-treatment with the novel PHD inhibitor FG-4497 improved the outcome from ischemic stroke. Our *in vitro* data indicate direct neuroprotection as well as indirect effects due to decreased formation of vasogenic edema by protecting the BBB integrity as underlying mechanisms. In line with our findings, other substances inhibiting PHD enzymatic activity such as deferoxamine mesylate (DFO), 3,4-dihydroxybenzoate (3,4-DHB) and DMOG have the potential to reduce brain injury upon cerebral ischemia in rodent models of stroke [1], [21], [22], [23], [24]. DFO, 3,4-DHB and DMOG act as competitive antagonists for co-factors of the PHD enzymes. While DFO is an iron chelator, 3,4-DHB and DMOG compete for 2-oxoglutarate [1], [21], [22], [23], [24]. However, as numerous enzymes rely on free iron availability and there are currently 50–60 known 2-oxoglutarate-dependent dioxygenases [25], an undesired interference with proteins other than PHDs by treatment with DFO, 3,4-DHB or DMOG is likely. Thus, development of novel drugs exhibiting an inhibitory potential limited to the enzymatic activity of PHDs appears indispensable to avoid not calculable risks during clinical use. This is further encouraged by our recent study demonstrating that neuron-specific inhibition of the single PHD isoform PHD2 is sufficient to attenuate acute neuronal loss in mice suffering from stroke [16].

### FG-4497 Induces HIF Pathway *in vitro* and *in vivo*

Overall, the present results provide clear evidence that FG-4497 induces the HIF signaling pathway in astrocytes and brain endothelial cells building up the BBB *in vivo*. Both cell types responded to FG-4497 treatment or lowered oxygen tension with a strong up-regulation of PHD2 and PHD3, whereas PHD1 was down-regulated in the presence of FG-4497. Similarly, Erez et al. reported that hypoxia or treatment with DFO suppressed PHD1 transcription which may involve transrepression by HIF-β but not HIF-α [26]. Induction of the main HIF repressors PHD2 and PHD3 is believed to limit the adaptive HIF response to tissue hypoxia or ischemia [27]. Additionally, FG-4497-induced stabilization of HIF-α correlated with up-regulation of the neuroprotective factors VEGF and Epo. Furthermore, application of FG-4497 via intraperitoneal route resulted in significant up-regulation of HIF-1α and induction of its target genes VEGF and Epo in brain tissue indicating the drug’s ability to cross the BBB. These findings are in accordance with the study of Schneider et al. showing induction of the HIF pathway in developing mouse brain upon short-term FG-4497 treatment [28].

### Protection of BBB *in vitro* and *in vivo*

BBB integrity is predominantly maintained by TJ between cerebral microvessel endothelial cells. TJ are located beneath the apical surface of adjacent endothelial cells and are composed of a combination of transmembrane (e.g. occludin, claudin-5) and cytoplasmic (e.g. ZO-1) proteins linked to an actin-based cytoskeleton allowing these junctions to form a tight seal [29]. Ischemic stroke causes loss of TJ protein localization and/or expression along the cellular membrane resulting in increased paracellular permeability leading to vasogenic edema formation and secondary brain damage [4]. In accordance, we could show that in brain endothelial cells the membrane localization of ZO-1 and occludin was disrupted under ischemic conditions *in vitro* as well as *in vivo*. Remarkably, pharmacological PHD inhibition by treatment with FG-4497 sustained the membrane localization of endothelial occludin and ZO-1, and was sufficient to reduce vasogenic edema formation during ischemic stroke. Similarly, Chen et al. reported that global genetic inactivation of PHD2 diminishes occurrence of cerebral edema in the early period after stroke [30]. It remains to be established whether attenuation of BBB breakdown by FG-4497 is HIF-dependent involving Epo and VEGF, or occurs via HIF-independent mechanisms.

A number of studies favor a role for VEGF and Epo in controlling vascular leakage. Beside others, the expression and subcellular localization of the junctional proteins occludin and ZO-1 is controlled by VEGF [31] and Epo [12], both secreted by astrocytes and endothelial cells and thus acting in an auto- and paracrine way.

VEGF is widely expressed in the central nervous system [32]. Apart from its neuroprotective effect [32], many studies have shown that it induces vascular permeability, both *in vitro* [33] and *in vivo* [34]. Concerning edema formation after stroke, VEGF seems to have a biphasic effect, promoting vascular leakage early after vessel occlusion, while showing no adverse effects on BBB permeability later on [35]. VEGF acts by rearrangement or increased degradation of TJ proteins, involving their phosphorylation [36], [37] or activation of matrix metalloproteinases [31], [38].

Epo, on the other hand, is mainly expressed by glial cells and neurons [39]. Contrary to VEGF, Epo is not only neuroprotective [6], [7] but also stabilizes BBB function, e.g. by VEGF antagonism [40] and conservation of TJ integrity [11], [12]. Thus, it can be anticipated that VEGF and Epo synergistically increase neuronal survival upon ischemic insult, while Epo in addition counteracts VEGF-induced hyperpermeability. Our results clearly imply a beneficial effect of PHD inhibition and thus HIF stabilization and increased transcription of VEGF and Epo. Simultaneous induction of VEGF and Epo resulted in neuroprotection (smaller infarct volumes) and reduced edema formation. Moreover, occludin and ZO-1 localization along the membrane of microvessel endothelial cells within the peri-infarct area was conserved under FG-4497 treatment. Consistent with that, Epo expression was higher under FG-4497 compared to control treatment in the ipsilateral hemisphere of the MCAO mice, underlining the role of Epo for compensating edema formation. However, it remains elusive that FG-4497 treatment of cerebral endothelial cells *in vitro* stabilizes tight junctions under ischemic conditions, although the PHD inhibitor induces VEGF, but fails to promote Epo expression at detectable level. One can speculate that PHD inhibition promotes unidentified mediators in an HIF-dependent or -independent manner that have the potential to counteract the disruption of tight junctions induced by VEGF activity. Notably, our *in vitro* approach demonstrating a direct cytoprotective effect of FG-4497 on neurons exposed to ischemic conditions confirms the hypothesis that brain tissue conservation by FG-4497 treatment is dependent on both direct and indirect neuroprotective mechanisms – the latter trough diminished vasogenic edema formation.

However, HIF-dependent mechanisms have been discussed controversially. Genetic or pharmacological alteration of HIF-activity resulted in the opposite. While neuron-specific HIF-1α deletion increased tissue damage after stroke in one study [21], it reduced ischemic damage in another [41]. Furthermore, HIF-1α inhibition by the chemical compound YC-1 has been shown to increase damage after stroke while simultaneously inhibiting ischemia induced vascular leakage, normally associated with smaller tissue damage [42]. Thus, the exact role of HIF-1 and its target genes for stroke outcome and prevention needs to be clarified.

In summary, our data indicate a therapeutic potential for the PHD inhibitor FG-4497 to prevent neuronal damage and vascular leakage after stroke. It might also reduce edema formation in other brain pathologies associated with vascular leakage such as brain injury or cerebral tumors. Furthermore, its neuroprotective potency makes FG-4497 an attractive candidate to treat various neurodegenerative diseases associated with vascular dysfunction and neuronal cell loss, e.g. Alzheimer’s disease [43], [44], [45].
